# Commentary: Artificial intelligence and precision medicine: a pilot study predicting optimal ceftaroline dosage for pediatric patients

**DOI:** 10.3389/frai.2026.1808575

**Published:** 2026-04-20

**Authors:** Hassan Nawaz Tahir, Ehtisham Haider, Shahnila Javed, Mursala Tahir, Yousaf Ali

**Affiliations:** 1 Community/Preventive Medicine, Department of Medical Education, Dawadimi College of Medicine, Shaqra University, Shaqra, Saudi Arabia; 2 Punjab Medical College, Faisalabad, Pakistan; 3 Liaquat National Hospital and Medical College, Karachi, Pakistan; 4 Department of Community Medicine, Liaquat National Hospital and Medical College, Karachi, Pakistan

**Keywords:** artificial intelligence, ceftaroline, dose optimization, pediatrics, precision medicine

Ceftaroline is a fifth-generation cephalosporin that is active against Gram-positive pathogens, including methicillin-resistant *Staphylococcus aureus*, as well as selected Gram-negative pathogens (Frasca et al., 2026). In pediatric practice, dose optimization is challenging because of variability in renal maturation, body composition, and drug clearance. Standard weight-based dosing regimens may therefore lead to underexposure or overexposure. In this context, individualized dosing strategies are increasingly being investigated.

We applaud Frasca et al. (2026), who pilot-tested the use of machine-learning (ML) models to optimize ceftaroline dosing in children. Their analysis revealed high alignment with pharmacokinetic targets when advanced ML strategies were used, particularly ensemble models such as Random Forest and XGBoost. The Neural Network model achieved a mean absolute error of 1.53 mg, while XGBoost achieved 2.04 mg in predicting individualized optimized doses (Optimized AMT). Therapeutic alignment was achieved in 91–94% of patients using ensemble models targeting a plasma concentration of 10 mg/L. The authors also applied Local Interpretable Model-agnostic Explanations (LIME) to enhance model interpretability. Given the complexity of antibiotic administration in infants, this exploratory study constituted an important proof-of-concept for data-driven dose individualization.

However, several methodological and conceptual issues warrant consideration to ensure appropriate interpretation of the findings and to inform future research (shown in Table 1).

**Table 1:** Structured thematic synthesis of key strengths and limitations in AI-guided pediatric ceftaroline dosing.

Theme	Description	Representative findings	Implications
AI-based dose individualization	Application of ML models to optimize ceftaroline dosing using pharmacokinetic data.	Ensemble ML models achieved high pharmacokinetic target attainment; explainability tools such as LIME were incorporated.	Demonstrates feasibility of data-driven precision dosing in pediatrics.
Pharmacokinetic Targets vs. clinical outcomes	Reliance on target plasma concentrations as surrogate endpoints.	No direct assessment of clinical outcomes such as efficacy, microbiological clearance, or toxicity.	Limits inference about real-world clinical benefit without outcome-level validation.
Validation strategy and overfitting risk	Model training and evaluation performed at pharmacokinetic-event level rather than patient level.	Multiple pharmacokinetic events from the same patient were treated as independent observations.	May inflate performance metrics and reduce generalizability; patient-level validation is needed.
Circularity of outcome definition	Optimized dose derived from internally generated pharmacokinetic targets.	Dose recommendations calculated using the same data structure that informed model inputs.	Raises concern for partial circularity and overestimation of predictive validity.
Uncertainty quantification	Lack of uncertainty estimates around individual dose recommendations.	Confidence intervals reported for global performance but not for patient-level predictions.	Limits clinical interpretability and safety assessment in pediatric decision support.
Population scope and generalizability	Study restricted to infants aged 2–24 months.	Findings discussed broadly in the context of pediatric dosing.	Risk of over-extrapolation across pediatric age groups with distinct pharmacokinetics.

First, the analysis assumed that achieving a predefined plasma concentration target would result in clinical benefit. Although pharmacokinetic target attainment is considered a surrogate endpoint, no clinical outcomes were measured, including treatment response, microbiological eradication, toxicity, or adverse events. In pediatric antibiotic therapy, exposure–response relationships are context-dependent and influenced by the site of infection, pathogen susceptibility, and host factors. Consequently, it remains unclear whether improved simulated target attainment would translate into improved clinical outcomes without outcome-level validation (Hartman et al., 2020; Riley et al., 2019).

Second, the model was developed and validated at the pharmacokinetic event level rather than at the patient level. Because multiple events originated from the same individuals, repeated measurements were treated as independent observations, creating a potential risk of overfitting. The algorithms may have learned patient-specific patterns rather than generalizable pharmacokinetic relationships. As a result, the reported performance metrics may partially reflect within-patient similarity rather than true external predictive power. Despite the use of internal validation, patient-level validation approaches, such as leave-one-patient-out cross-validation, would enhance generalizability (Varoquaux and Colliot, 2023).

Third, the optimized dose modeled in the study was not a clinical endpoint but a derived variable calculated to minimize deviation from a predefined concentration target. This target was derived from the same pharmacokinetic framework that informed the model inputs. This introduces a potential element of circularity, whereby models optimize internally generated targets rather than externally validated therapeutic outcomes. Strong internal performance may therefore indicate internal consistency rather than real-world predictive validity (Varoquaux and Colliot, 2023).

Fourth, although the authors reported confidence intervals for overall performance measures, they did not quantify uncertainty surrounding individual dose recommendations. In pediatric pharmacotherapy, dosing errors can be asymmetric and potentially severe. Clinical decision support tools require not only point estimates but also measures of prediction uncertainty at the individual patient level. Providing uncertainty intervals for recommended doses would improve clinical interpretability and safety assessment (Verhaeghe et al., 2022).

Lastly, the study population was restricted to infants aged 2–24 months; however, conclusions were discussed within the broader context of pediatric dosing. Developmental pharmacokinetics differs substantially among neonates, infants, children, and adolescents. Models derived from infants cannot be assumed to generalize across the entire pediatric age spectrum. Explicitly limiting inference to the studied age group would prevent overextrapolation and enhance scientific precision (Ilic, 2025).

In summary, the article by Frasca et al. represents a valuable exploratory study on the use of explainable machine learning in pediatric dose optimization. Addressing concerns related to clinical outcome validation, patient-level generalization, potential circularity in target definition, incorporation of individual-level uncertainty estimates, and clearer population boundaries will be essential for translating this promising approach into safe and clinically meaningful practice.

## References

[B1] Frasca M. Gazzaniga G. Graziosi A. De Nicolo V. De Giacomo C. Martinelli S. . (2026). Artificial intelligence and precision medicine: a pilot study predicting optimal ceftaroline dosage for pediatric patients. Front. Artif. Intell. 8:1702087. doi: 10.3389/frai.2025.1702087 41626576 PMC12856755

[B2] Hartman S. J. F. Brüggemann R. J. Orriëns L. Dia N. Schreuder M. F. de Wildt S. N. . (2020). Pharmacokinetics and target attainment of antibiotics in critically ill children: a systematic review of current literature. Clin. Pharmacokinet. 59, 173–205. doi: 10.1007/s40262-019-00813-w 31432468 PMC7007426

[B3] Ilic K. (2025). Biomarkers as surrogate endpoints in drug development: finding their right place. Clin. Ther. 47, 944–949. doi: 10.1016/j.clinthera.2025.07.026 40973597

[B4] Riley R. D. Snell K. I. Ensor J. Burke D. L. Harrell F. E. Jr. Moons K. G. . (2019). Minimum sample size for developing a multivariable prediction model: PART II - binary and time-to-event outcomes. Stat. Med. 38, 1276–1296. doi: 10.1002/sim.7992 30357870 PMC6519266

[B5] Varoquaux G. Colliot O. (2023). “Evaluating machine learning models and their diagnostic value,” in Machine Learning for Brain Disorders, ed. O. Colliot (New York, NY: Humana), 601–630. doi: 10.1007/978-1-0716-3195-9_20 37988512

[B6] Verhaeghe J. Dhaese S. A. M. De Corte T. Vander Mijnsbrugge D. Aardema H. Zijlstra J. G. . (2022). Development and evaluation of uncertainty quantifying machine learning models to predict piperacillin plasma concentrations in critically ill patients. BMC Med. Inform. Decis. Mak. 22:224. doi: 10.1186/s12911-022-01970-y 36008808 PMC9404625

